# Cronkhite-Canada Syndrome Masquerading as Inflammatory Bowel Disease

**DOI:** 10.14309/crj.0000000000001090

**Published:** 2023-09-05

**Authors:** Justin Wen Hao Leong, Lai Mun Wang, James Weiquan Li, Tiing Leong Ang, Boon Eu Andrew Kwek, Jeannie Peng Lan Ong

**Affiliations:** 1 Department of Gastroenterology and Hepatology, Changi General Hospital, Singapore; 2 Department of Laboratory Medicine, Changi General Hospital, Singapore

**Keywords:** gastrointestinal polyposis, Cronkhite-Canada syndrome, inflammatory bowel disease

## Abstract

Cronkhite-Canada syndrome (CCS) is a rare nonhereditary gastrointestinal polyposis syndrome. We illustrate a case with clinical presentation of dysgeusia, chronic diarrhea and weight loss, and endoscopic features of diffuse gastric mucosa nodularity with circumferential nodular pancolitis and a solitary colonic polyp initially mimicking inflammatory bowel disease. After multidisciplinary discussion, the diagnosis of CCS was made. The patient received steroids with resultant clinical, endoscopic, and histological improvement. We discuss the treatment and risk of neoplasia in CCS.

## INTRODUCTION

Cronkhite-Canada syndrome (CCS) is a rare nonhereditary gastrointestinal polyposis syndrome first described in 1955 by Cronkhite Jr and Canada.^1^ It is characterized by unique features of chronic diarrhea, vomiting, dysgeusia, onychodystrophy, alopecia, and hyperpigmentation.^2,3^ The estimated incidence of CCS is 1 per million, with a mean age of 63 years and male predominance.^3^ Complications include malnutrition from protein-losing enteropathy, gastrointestinal bleeding, and increased risk of gastrointestinal cancer.^3,4^ CCS carries a mortality rate of up to 50% in previous series.^5^

## CASE REPORT

**Figure 1.**
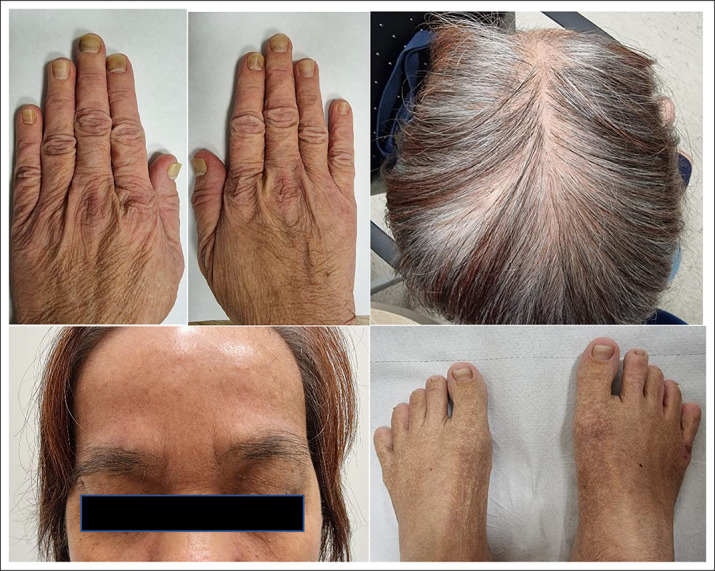
Clinical features of Cronkhite-Canada syndrome. Top left: Bilateral onycholysis; top right: alopecia; bottom left: periorbital hyperpigmentation; bottom right–xerosis: fissuring and hyperpigmentation.

**Figure 2.**
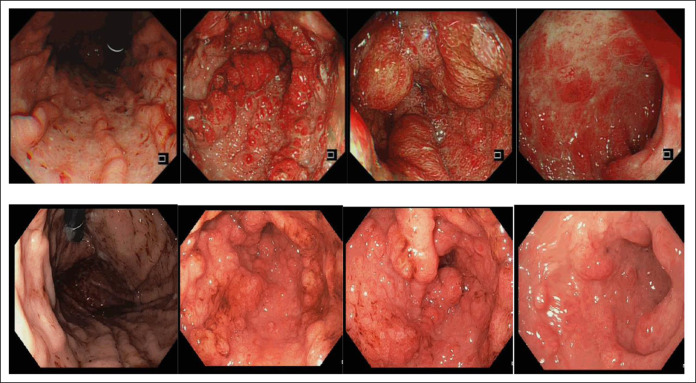
Top row: index oesophagogastroduodenoscopy (OGD) showing edematous and nodular mucosa at the antrum and duodenal bulb with multiple hyperplastic polyps. Bottom row: Repeat OGD at 4 months showing regression of previously seen polyps.

**Figure 3.**
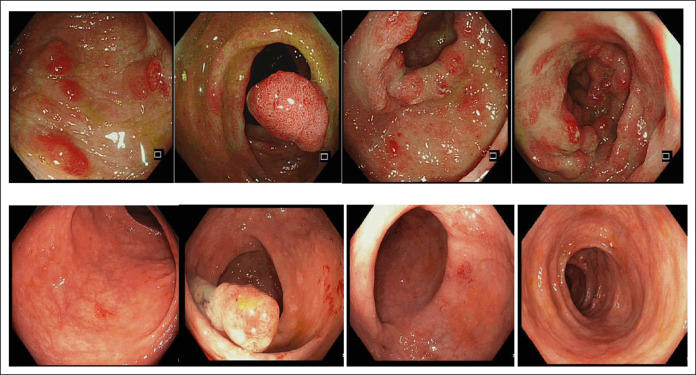
Top row: index Colonoscopy showing congested and edematous mucosa with dense nodule hyperplasia. Bottom row: repeat colonoscopy showed marked improvement of previously seen confluent nodular and circumferential colitis.

**Figure 4.**
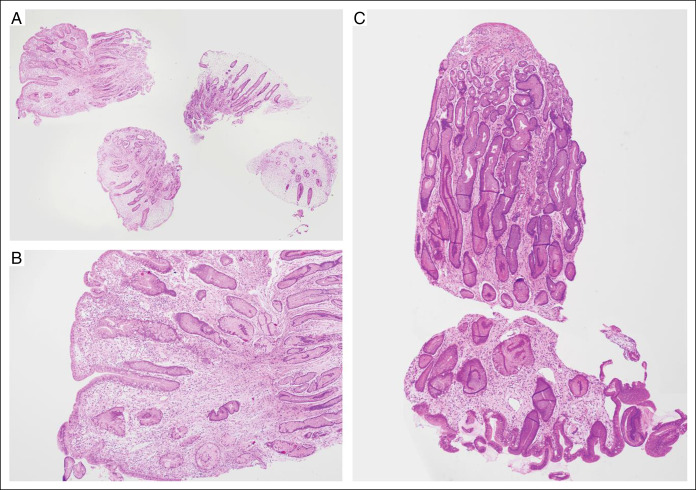
(A) Haematoxylin & eosin (H&E)-stained slide of gastric corpus biopsy representing endoscopic nodular changes at low magnification 20× showing multiple fragments of gastric mucosa with hyperplastic foveolar epithelium and oedematous lamina propria and is without parietal and chief cells. (B) Higher magnification 200× confirms lack of dysplasia and malignancy. Overall, the features are consistent with multiple hyperplastic polyps. (C) Hyperplastic polyps are still present on the repeat oesophagogastroduodenoscopy with biopsy (H&E, 200×).

Our patient is a 58-year-old Chinese woman who first presented with dysgeusia of 3 weeks’ duration. Over the next 2 months, she developed vomiting, loose stools, numbness over bilateral hands and feet, and weight loss of 6 kg with increased pigmentation and thickening over her eyes, hands, and feet with hair loss. Clinical examination revealed xerosis and onycholysis with periorbital hyperpigmentation and alopecia (Figure 1). Laboratory results showed hemoglobin 15.5 g/dL (12–16 G/DL), C-reactive protein 31.9 mg/L (<3.0 mg/L), albumin 29 g/L (40–51 G/L), and zinc 832 μG/L (724–1,244 μG/L). Fecal calprotectin was 765 µg/g (<80 µg/g). Esophagogastroduodenoscopy showed diffuse gastric mucosal hyperemia, edema, and nodular changes (Figure 2), which were proven to be hyperplastic polyps histologically (Figure 3). *Helicobacter pylori* was negative. Colonoscopy revealed a confluent circumferential nodular colitis throughout the colon with a single 1 cm sessile polyp in the transverse colon (Figure 4).

**Figure 5.**
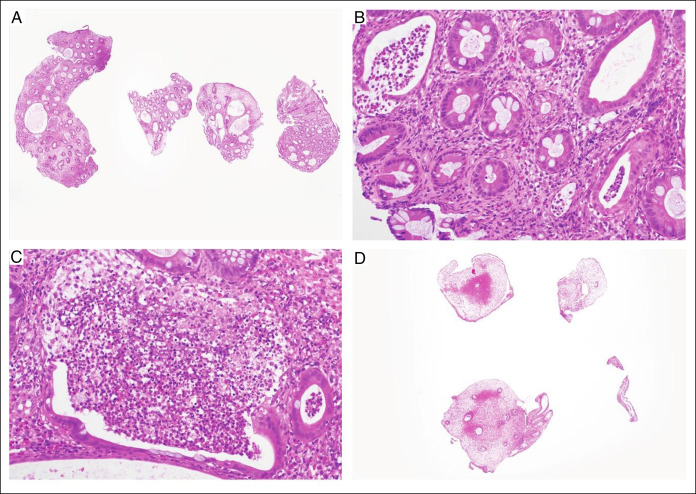
(A) Haematoxylin & eosin (H&E)-stained slide of the rectal biopsy showing diffuse crypt architectural distortion (20×) accompanied by (B) patchy mild to moderate increase in chronic inflammatory cell density within the lamina propria and crypt abscesses (200×). (C) Higher magnification demonstrates cryptitis and crypt abscess formation with crypt disruption (400×). Similar inflammatory bowel disease like-changes were also seen in multiple site colonic biopsies taken from the caecum to the sigmoid colon. (D) H&E section (20×) of the 1 cm polyp at initial presentation showing oedematous inflamed colonic mucosa without dysplasia or malignancy, in keeping with an inflammatory pseudopolyp.

Histology showed features reminiscent of chronic inflammatory bowel disease with mild ileitis and continuous diffuse crypt architectural changes, cryptitis, and crypt abscess formation extending from the cecum to the rectum (Figure 5). The chronic inflammatory cell was moderately increased in the lamina propria but lacked basal plasmacytosis. In the setting of active chronic proctocolitis, the histology of the single polyp was interpreted as an inflammatory pseudopolyp (Figure 5).

In view of the clinical symptoms of dysgeusia and diarrhea and associated ectodermal symptoms with atypical endoscopic features of colitis in the absence of classical polyposis, her case was discussed at a multidisciplinary meeting. The final diagnosis was CCS based on the constellation of clinical and endoscopic features, although the diagnosis is difficult in the absence of classical polyposis.

**Figure 6.**
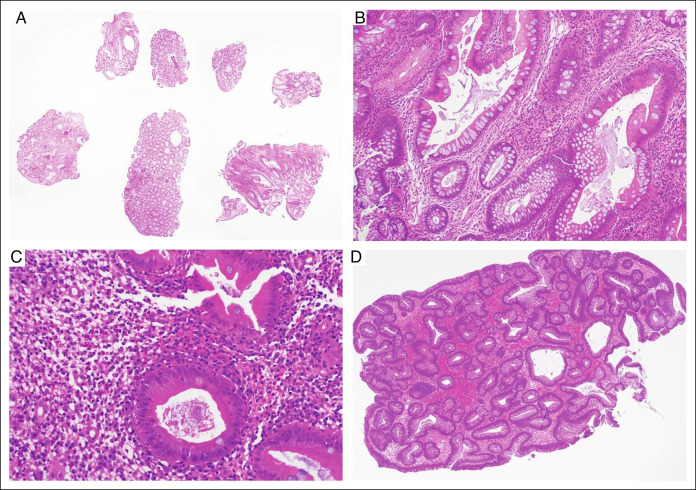
Follow-up colonic biopsies after starting steroid treatment. (A) Haematoxylin & eosin (H&E)-stained slide of the rectal biopsy showing persistence of crypt architectural distortion in all fragments (20×). (B) Higher magnification of the distorted crypts with moderate increase in chronic inflammatory cell infiltrate present within the lamina propria (200×). (C) An improvement in activity is seen with cryptitis only. The crypt lumina contains debris without neutrophilic abscess (400×). (D) H&E section (40×) of the 1 cm polyp at initial presentation displays histological features of tubular adenoma with low grade dysplasia.

She was treated with high-dose steroid therapy (prednisolone 40 mg once daily for 10 days, followed by 30 mg for 2 weeks and subsequently 20 mg for 4 weeks) and mesalamine with caloric and protein supplementation, achieving significant improvement in her symptoms. However, on tapering off her prednisolone dose to 15 mg once daily after 8 weeks of high-dose steroids, recrudescence of symptoms occurred. Prednisolone was escalated with resultant improvement. At 4 months of treatment, the patient achieved clinical response with improvement of appetite, weight gain, improvement in diarrhea, and normalization of serum albumin. Repeat esophagogastroduodenoscopy showed gastric hyperplastic polyps (Figure 4), and repeat colonoscopy demonstrated marked improvement of colitis diagnosed previously (Figure 2). Severity of activity improved, but features of chronicity remained on her colonic biopsies histologically (Figure 6). A solitary 10 mm sessile polyp at the transverse colon was resected, with histology showing tubular adenoma with low-grade dysplasia.

## DISCUSSION

CCS is a rare syndrome with diagnosis is made based on a combination of history, physical examination, endoscopy with findings of gastrointestinal polyposis, and histology. Differential diagnoses for diffuse hamartomatous polyposis include Peutz-Jeghers syndrome, juvenile polyposis syndrome, and Cowden syndrome, although the extraintestinal manifestations of dermal hyperpigmentation, onychodystrophy, and alopecia are unique to CCS.^6^

The underlying pathogenesis remains obscure and has been postulated to have an autoimmune component, with positive IgG4 immunostaining and the efficacy of immunosuppression in treatment giving credence to this.^7,8^

Steroid therapy and nutritional support are the mainstays of medical treatment, with a large majority of patients showing clinical improvement with resolution of gastrointestinal symptoms and regression of skin changes including hyperpigmentation and onycholysis.^3^ Too rapid tapering of steroids can be associated with early relapse, and it is suggested that the steroid dose be tapered only after endoscopic confirmation of regression of polyposis. Recurrences are not uncommon, but often respond to corticosteroid retreatment, as in our patient.

The optimum treatment of CCS is currently unknown with no consensus guidelines, particularly in patients who are steroid-refractory. Steroid-sparing therapies including azathioprine, cyclosporine A, and anti-TNF-α have been used in such cases to induce or maintain remission.^9–11^ Isolated case reports have described the eradication of *H. pylori* in these patients leading to complete remission; however, no clear association has been established on its prognostic significance.^12,13^

Finally, the risk of neoplasia in CCS is controversial with questions surrounding the carcinogenic potential of the disease. Watanabe et al reported a prevalence of 10%–20% of gastric or colon cancer among a cohort of Japanese patients.^3^ The majority were adenocarcinoma with histopathological findings consistent with the adenoma-carcinoma sequence, although it is not possible to discount the increased malignant potential through inflammation-induced mutation. Owing to the rarity of the disease, optimal screening protocols have not been defined, although annual surveillance has been practiced.

The diagnosis of CCS can be elusive in patients who do not present with classical endoscopic features. However, given the significant symptom burden and serious complications if left untreated, it is imperative that CCS be considered as a differential, particularly in the appropriate clinical context. The mainstay of treatment is steroid therapy and nutritional support with clinical and endoscopic remission as therapeutic goals. Finally, we raise the awareness of neoplasia in CCS, and patients should be monitored with regular endoscopic surveillance.

## DISCLOSURES

Author contributions: JWH Leong collaborated in data collection, literature search, and writing of the manuscript. LM Wang collaborated in the critical revision of the manuscript and pathology images. JW Li, TL Ang, BEA Kwek, and JPL Ong collaborated in critical revision of the manuscript. JPL Ong is the article guarantor.

Financial disclosure: None to report.

Informed consent was obtained for this case report.
